# Substructure-based neural machine translation for retrosynthetic prediction

**DOI:** 10.1186/s13321-020-00482-z

**Published:** 2021-01-11

**Authors:** Umit V. Ucak, Taek Kang, Junsu Ko, Juyong Lee

**Affiliations:** 1 Division of Chemistry and Biochemistry, Department of Chemistry, Kangwon National University, Chuncheon, South Korea; 2 Center for Neuro-Medicine, Brain Science Institute, Korea Institute of Science and Technology, Seoul, South Korea; 3 Arontier co., Seoul, South Korea

**Keywords:** Retrosynthesis planning, Neural machine translation, Seq-to-seq, Attention

## Abstract

With the rapid improvement of machine translation approaches, neural machine translation has started to play an important role in retrosynthesis planning, which finds reasonable synthetic pathways for a target molecule. Previous studies showed that utilizing the sequence-to-sequence frameworks of neural machine translation is a promising approach to tackle the retrosynthetic planning problem. In this work, we recast the retrosynthetic planning problem as a language translation problem using a template-free sequence-to-sequence model. The model is trained in an end-to-end and a fully data-driven fashion. Unlike previous models translating the SMILES strings of reactants and products, we introduced a new way of representing a chemical reaction based on molecular fragments. It is demonstrated that the new approach yields better prediction results than current state-of-the-art computational methods. The new approach resolves the major drawbacks of existing retrosynthetic methods such as generating invalid SMILES strings. Specifically, our approach predicts highly similar reactant molecules with an accuracy of 57.7%. In addition, our method yields more robust predictions than existing methods.

## Background

Although knowledge in organic chemistry has accumulated over decades, designing an efficient synthetic route for a target molecule remains a crucial task in organic synthesis [1]. The retrosynthetic approach suggests a logical synthetic route to generate a target molecule from a set of available reactants and reagents [2–4]. This approach is both iterative and recursive in nature since a sequential computation of retrosynthetic transformation is required. Retrosynthetic transformation occurs recursively until much simpler and commercially available molecules are identified.

Computational retrosynthetic analysis initially formalized in 1969 by Corey and Wipke in an algorithmic manner [5]. The algorithm considers all possible disconnections with known reaction types, which reduce the complexity of a product and progress until chemically reasonable pathways are identified. Such disconnections were based on handcrafted minimal transformation rules known as reaction templates [5–7]. Manual encoding of those transformation rules necessitates deep chemical expertise and intuition. Manual management of synthetic knowledge is a highly complicated task considering a large number of transformation rules (> 10,000) that must be hand-coded [8–11]. Furthermore, being dependent on reaction templates potentially limits prediction accuracy, particularly if a reaction is outside of the template domain. Later studies offer valuable help to chemists in finding better routes faster by enabling automated extraction of reaction templates [12–18]. However, they do not address the above-mentioned limitations inherited from their precedents. Computer-aided synthesis planning has been well summarized in many recent reviews [19–24].

Reaction predictor developed by Kayala et al. [25, 26] was the first template-free approach. It was a mechanistic level of strategy that merges the idea of rule-based modeling and machine learning within its framework. Jin et al. [27] proposed a novel template-free, entirely data-driven approach based on the Weisfeiler-Lehman networks [28]. Both approaches provide end-to-end solutions to generate candidate products. Theoretical findings provided by Cadeddu et al. [29] have further motivated the development of other template-free methods for the forward- or retro-reaction prediction tasks using various types of neural machine translation (NMT) architectures [30–38]. Based on an explicit analogy between sentences in a language corpus and molecules in a chemical corpus, i.e. chemical space, Cadeddu et al. showed that the rank-frequency distributions of substructures as the building blocks of molecules are similar to those of words in a natural language corpus. This verification implies that the concepts of linguistic analysis are readily applicable to tackle the problems of forward- and retro-reaction prediction. In this context, a retrosynthetic prediction is appropriate for applying the sequence-to-sequence framework [39–41] of machine translation.

Sequence-to-sequence learning uses a recurrent neural network (RNN) layer to map a source sequence of an arbitrary length into a fixed dimensional context vector consisting of real numbers. The context vector contains information about the syntactic and semantic structure of the source sequence. In connection with this RNN layer, another RNN decodes the context vector to a target sequence. In this regard, the two RNN units together act like a pair of encoder–decoder system. Sutskever et al. [41] showed that long short-term memory (LSTM) [42]-based architectures can solve general sequence-to-sequence problems because of their ability to handle long-range relations in sequences. Liu et al. [34] proposed the first multi-layered LSTM-based sequence-to-sequence model for retrosynthetic prediction. Its gated recurrent unit (GRU) [39] variant was proposed by Nam and Kim [32] for the forward reaction prediction.

Recently, the best performing NMT models include an attention mechanism [40, 43] as a part of their neural architectures to enhance their performances on longer sentences [27, 32–34]. There are also retrosynthetic predictors built on the Transformer architecture [31, 37, 44–46], based solely on the attention mechanism. Encoder–decoder models, especially once an attention mechanism is introduced, all employ similar strategies to handle a translation task. The SMILES representations of molecular structures are typical inputs for the sequence-to-sequence based models. However, none of the previously reported models has focused on translation at a substructural, fragment, level.

In this paper, we propose a template-free approach for retrosynthetic reaction prediction by learning the chemical change at a substructural level. Our approach represents a molecule as a sentence based on a set of substructures corresponding to a word by using the MACCS keys [47]. We also present a unique tokenization scheme that properly eliminates problematic issues originate from SMILES-based tokenization. Our model consists of bidirectional LSTM cells [48], and is trained in a fully data-driven and end-to-end fashion without prior reaction class information. We thoroughly discuss all the aspects of our methodology, including dataset and descriptor curation steps. Evaluation results are presented based on three datasets derived from the United States Patent and Trademark Office (USPTO) reaction dataset [49].

This paper is organized as follows. In "Method" section, we suggest a new way of tokenization followed by curation together with the analysis of the dataset and descriptor. We briefly describe the model architecture and evaluation procedure for accuracy calculations. In "Results and discussion" section, the results of a set of translation experiments are discussed with an emphasis on the benefits of the MACCS key-based molecular representation. Finally, the strengths and limitations of our approach are concluded in "Conclusion" section.

## Method

### Dataset

In this study, we used the filtered US patent reaction dataset, USPTO, which is obtained with a text-mining approach [49, 50]. Schwaller et al. [33] eliminated the duplicated reaction strings in the dataset without atom-mapping. They also removed 780 reactions due to SMILES canonicalization failures with RDKit [51]. The inherent limitation of the data is that the vast majority of entries are single product reactions. Thus, only single product cases corresponding to 92% of the dataset are used in this study.

The SMILES line notation [52] represents molecular structures as a linear sequence of letters, numbers, and symbols. Hence, from a linguistic perspective, SMILES can be regarded as a language with grammatical specifications. However, in our approach, molecules are represented as a set of fragments using the MACCS keys consisting of 166 pre-defined substructures [47]. This binary bit-based molecular descriptor converts a molecule into a 166 bit vector, in which each bit indicates the presence of a feature taken from a predefined dictionary of SMARTS patterns [53].

### Descriptor curation

**Fig. 1:**
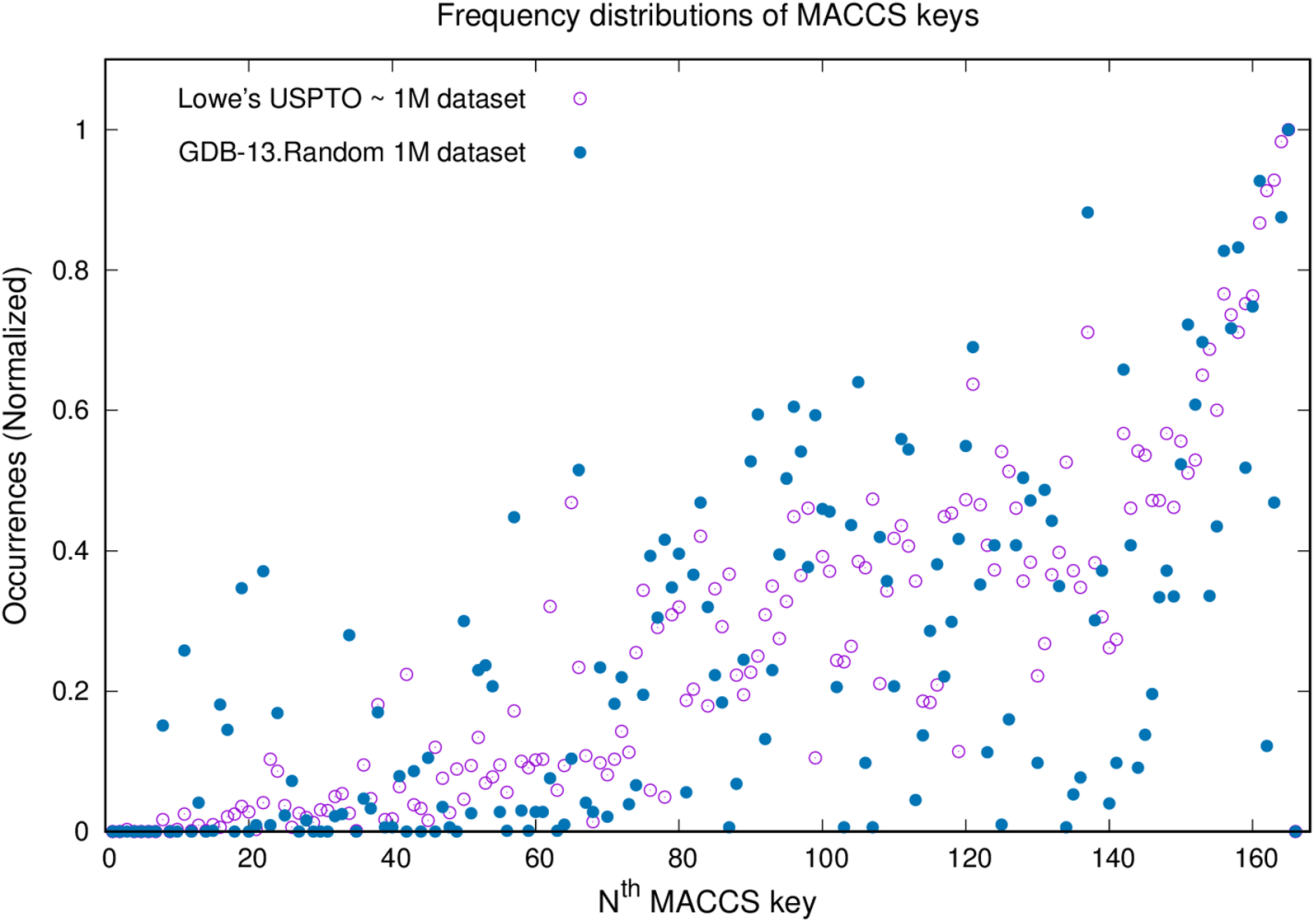
Descriptor curation based on the rate of occurrences. Filtered US patent reaction dataset and 1 million randomly sampled drug-like small molecules as a subset of the enumerated database (GDB-13) are compared to investigate the MACCS keys probability distribution profiles

In our approach, a molecule is represented as a set of fragments using the MACCS keys. The number of occurrences of each MACCS key in our dataset was investigated. Also, we compared the results obtained for 1 million randomly sampled drug-like small molecules, a subset of the Generated Data Base-13 (GDB-13) consisting of 975 million molecules [54, 55]. Figure 1 shows the normalized frequency distributions of the MACCS keys on both databases. A direct pairwise comparison rationalizes reducing the number of MACCS keys (Fig. 1). In this study, five keys that never occurred and nine keys that are not frequently observed in the USPTO database are omitted. Based on the comparison, additional 26 keys that are never or hardly ever observed in the GDB-13 database are also excluded.

The molecules belong to different compound databases, such as drug-like or natural products, exhibit different characteristics in their fingerprint profiles. Thus, we narrowed our analysis to drug-like molecules and modified our fingerprint representation accordingly by tuning it with 1 million drug-like small molecules in GDB-13. Removing redundant keys based on the occurrence analysis has apparent advantages. It shortens the lengths of source and target sentences and provides a better rank distribution of the keys used in the translation process. In our approach, every molecule is represented by 126 MACCS keys, which are able to represent 98% of the 1 million randomly sampled subset of GDB-13 adequately. In machine translation tasks that chemists are dealing with, source and target molecules are placeholders corresponding to reactants and products interchangeably. The selection is dependent on the intended analysis. For a retrosynthetic prediction task, source and target sentences refer to *products* and *reactants*, respectively.

### Reaction preprocessing

**Fig. 2:**
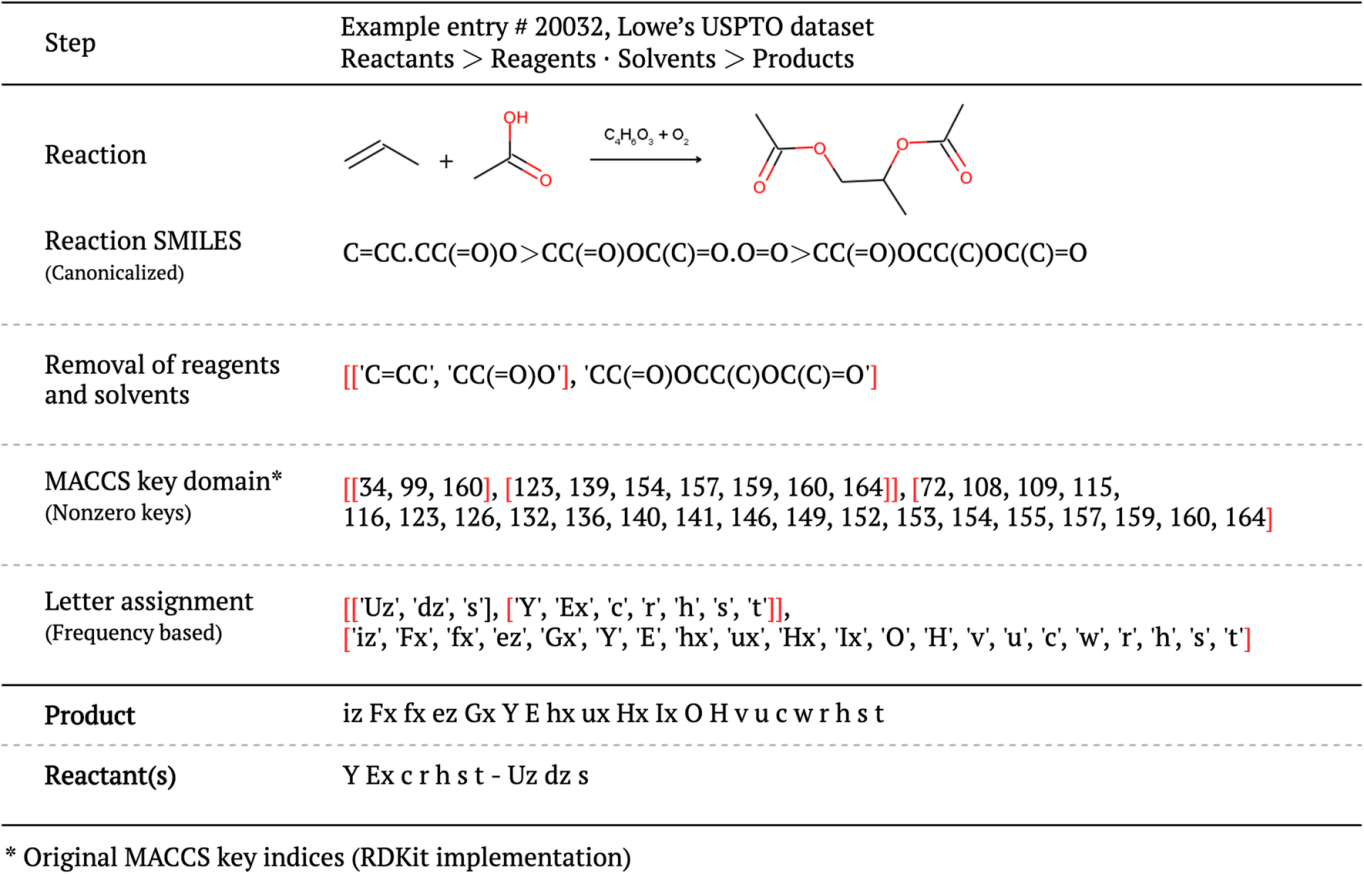
Data preparation procedure to obtain product and reactant sentences for a retrosynthetic prediction task

Our model considers only the non-zero indices of curated MACCS keys. English letters were assigned to the ranked non-zero MACCS keys based on their *ranks of frequencies* to form unique artificial “words”. This further encoding transforms product and reactant sentences into the frequency-based sorted version of the lettered keys, which imply position-wise information of the words, and make our scheme suitable for using the sequence-to-sequence architecture. Single-lettered words were generated using the upper- and lower-cases of the most frequent 21 letters in English. Double-lettered words were constructed by adding “x” and “z” for every 42 single letters, which allowed us to cover all 126 MACCS keys. Thus, our lettered fragment vocabulary has a fixed length of 126. The generation process of an example product–reactant pair is illustrated in Fig. 2. The same procedure was applied to all reactions of the dataset. The complete mappings of the MACCS keys to artificial words are listed in Additional file 1.

The MACCS non-zero indices serve as good tokens and inputs for an LSTM model. The model further encodes the products and reactants into “language representation” by assigning one or two letters to each index in the MACCS keys. Applying further encoding is efficient, particularly given the relatively small size of curated MACCS keys. It gives a rank-order, enhances readability, and provides visual comprehension.

### Reaction dataset curation

The product–reactant pair dataset was further curated before being processed by our translation machine. After representing every molecule with the 126 truncated MACCS keys, a series of filters were applied to remove identical product–reactant pairs and internal twins. Internal twins are the pair of data entries whose product and reactant sentences are identical. They appeared whenever the chemical changes were beyond the sensitivity of our MACCS key-based representation. Because we associate molecules with MACCS keys to operate on a substructural subspace, a certain amount of information is lost. Our preprocessing procedure resulted in 5748 internal twins, and they are removed from our dataset. In addition, the reactions with three or more reactants were excluded. The length of the longest pair was set to 100 to avoid lengthy fragment sequences, as shown in Additional file 2: Figure S1.

**Fig. 3:**
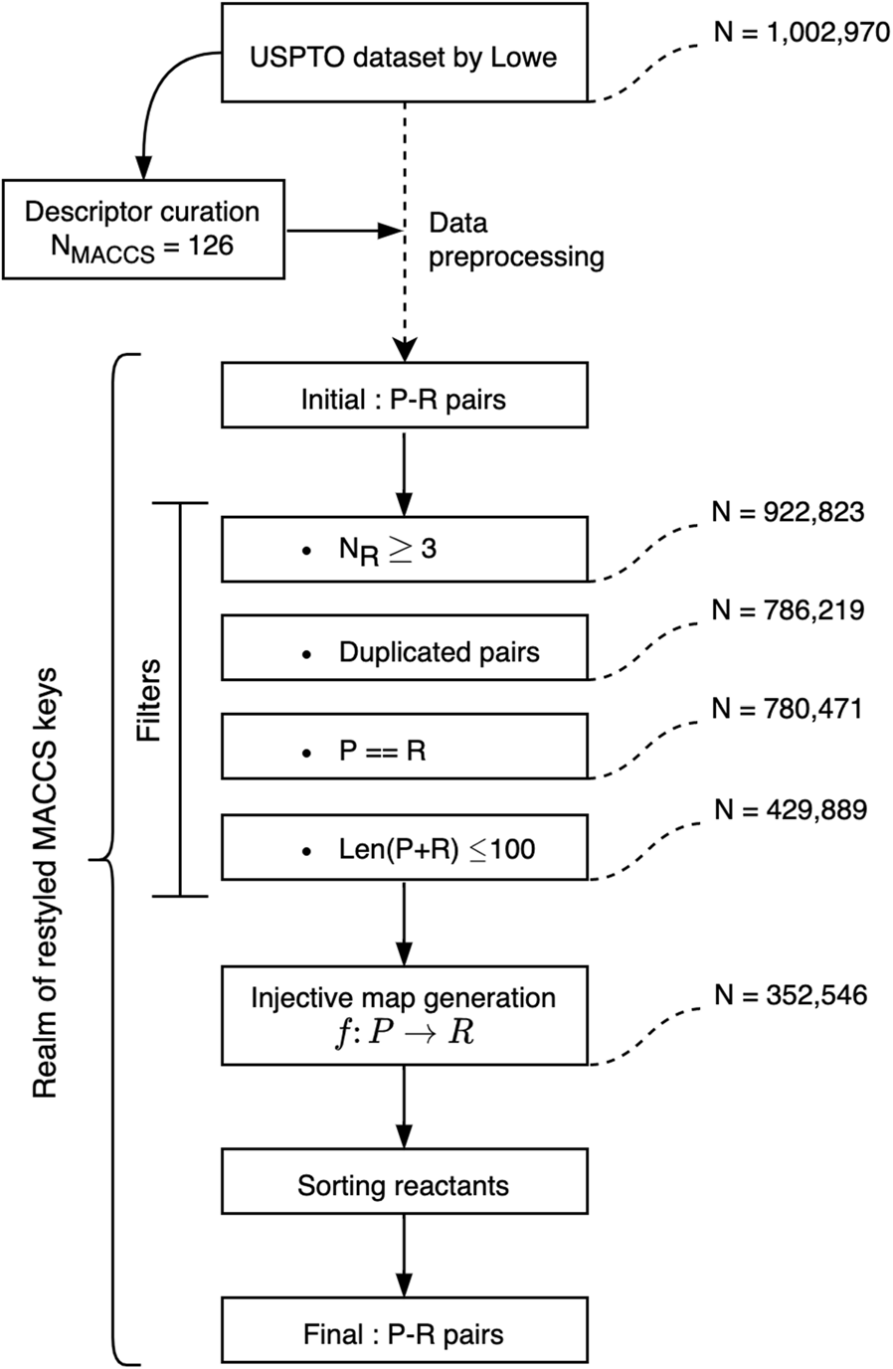
Dataset curation process and obtaining training/test pairs.* P* Product,* R* Reactant. Details to the different steps are given in the text

The product–reactant pairs were then put into an injective map generator to guarantee one-to-one correspondence between product and reactant sentences. If a reactant sentence is composed of two reactants, we sorted them in descending order according to their sequence length. Reactants were separated by the “–” sign. The curated dataset, containing a total of 352,546 product–reactant pairs, was further subdivided by the number of the reactant molecules in each pair into two disjoint subsets: single reactant and double reactant datasets. Organizing the dataset in this manner was essential to assess model performance independently. These data sets are freely available online, and curation steps along with the dataset sizes are summarized in Fig. 3.

### Model architecture

Our sequence-to-sequence neural network comprises two bidirectional LSTMs: one for an encoder and the other for a decoder. Besides, we used unidirectional LSTMs to quantify the improvement in model’s performance with the use of bidirectional LSTMs. The encoder and decoder layers were connected through Luong’s global attention mechanism [56], which captures non-local relations between all elements of source sequences. The attention mechanism allows neural networks to focus on different parts of a source sentence, and to consider non-linear relationships between words during a training process. The global attention mechanism used in this study, in essence, is similar to the first attention mechanism suggested by Bahdanau et al. [40], for machine translation tasks. The global approach focuses the “attention” on all the words on the source sentence to compute a global context vector for each target word at each time step in the decoder unit. Therefore, the global context vector represents the weighted sum over all the source hidden states. This context information leads to improved prediction accuracy.

### Training details

Our curated datasets were randomly split into 9:1 to generate training and testing sets. The validation sets were randomly sampled from training sets (10%). The word embeddings were used to represent lettered fragments in the vocabulary. After the embedding layer was created, a trainable tensor holding 126-dimensional fixed-length dense vectors was randomly initialized. A method of embedding class then accessed the embedding of each word through a lookup on the tensor. We used the stochastic gradient descent algorithm [57] to train all parameters of the encoder–decoder model. The cross-entropy function was used as a loss function.

For each dataset, we performed a series of tests within the range of hyper-parameter space as described in Additional file 8: Table S1, to achieve optimal performance. Based on the preliminary experiments, we generated an encoder and a decoder with two Bi-LSTM layers containing 2000 hidden units at each layer. A dropout layer with a dropout rate of 0.1 was included following the hidden layer to avoid overfitting. To avoid a potential exploding gradient problem, we introduced gradient clipping [58] to guarantee that the norm of the gradients did not exceed a threshold (0.25) during backpropagation. The initial learning rate was set to 4.0, and it decayed with a factor of 0.85 every three epochs [33].

With these hyper-parameters, the average training speed was approximately 3300 words per second, with a batch size of 64 on a single NVIDIA RTX 2080Ti GPU card. Larger batch sizes were not tested due to memory constraints, which likewise apply to the hidden layer’s size. We trained our models for a minimum of 30 epochs, and each epoch took about 2 h for the curated dataset consisting of 320 K sentence pairs. The details of our key hyper-parameters are available in Additional file 8: Table S1.

Our model was implemented in Python version 3.6.8 together with PyTorch [59] version 1.3.0. The open-source RDKit module version 2020.03.1 [51] was utilized to obtain MACCS keys and similarity maps [60].

### Evaluation procedure

Association coefficients such as Tanimoto, Sörensen–Dice, and asymmetric Tversky indexes are considered efficient similarity measures for structural similarity benchmarks, and thus they are widely used. To evaluate the performance of our retrosynthetic model, the Tanimoto coefficient was selected as a similarity metric, which is identified as one of the best metrics to compute structural similarity [61]. Pairwise similarities between the predicted sequences and ground truth of all test molecules were calculated. Tanimoto coefficient ($T_c$) measured between two chemical structures have a value between 0 and 1. The coefficient is zero if molecules share no common fragments while identical molecules have a Tanimoto coefficient of unity. Though these are the cases for the two ends of the Tanimoto similarity metric, there is no single criterion that defines similar and non-similar molecules. We defined three threshold values (0.50, 0.70, and 0.85) to assess the quality of translation experiments. The similarity between predicted and ground truth sentences was computed at the end of each epoch for every pair appear in the validation set using the Tanimoto similarity measure (Eq. 1).

1 $$\begin{aligned} T_{c}\small {(\mathbf {R},\mathbf {P})} = \frac{{\displaystyle {\sum _{i}}}{R}_{i}{P}_{i}}{{\displaystyle {\sum _{i}}}{\left( {R}_{i}\right) }^2+{\displaystyle {\sum _{i}}}{\left( {P}_{i}\right) }^2-{\displaystyle {\sum _{i}}}{R}_{i}{P}_{i}}. \end{aligned}$$

**Table 1:** The possible pairs between predicted sequences and ground truths are presented

Ground truth	Predictions	List of possible pairs
P → R$_{A}$ + R$_{B}$	P → P$_{A}$ + P$_{B}$	[($\mathbf{R} _{A}{} \mathbf{P} _{A}$; $\mathbf{R} _{B}{} \mathbf{P} _{B}$), ($\mathbf{R} _{A}{} \mathbf{P} _{B}$; $\mathbf{R} _{B}{} \mathbf{P} _{A}$)]
	P → P$_{C}$	[$\mathbf{R} _{A}{} \mathbf{P} _{C}$, $\mathbf{R} _{B}{} \mathbf{P} _{C}$]
P → R$_{C}$	P → P$_{A}$ + P$_{B}$	[$\mathbf{R} _{C}{} \mathbf{P} _{A}$, $\mathbf{R} _{C}{} \mathbf{P} _{B}$]
	P → P$_{C}$	[$\mathbf{R} _{C}{} \mathbf{P} _{C}$]

The similarity of each pair is computed with the Eq. 1

Our machine yields predictions either with one or two reactants as all reactions are contained in the combined dataset. There are thus multiple possibilities for comparing predicted sequences with ground truths. The potential pairs for evaluation corresponding to the number of reactants are listed in Table 1. Tanimoto similarities between all possible pairs of predicted sequences and ground truths were calculated. Then, the pair(s) with the highest similarity was selected based on the assumption that more similar structures are more likely to be matched.

## Results and discussion

### Prediction accuracy

The performance of our model was assessed based on three datasets: single reactant, double reactant, and the combined test set. Evaluation results on the test sets are summarized in Table 2. The quality of predictions of each test dataset is expressed in terms of pairwise Tanimoto similarity values. We introduced three criteria for evaluating the success rates of our translation models: (1) the number of exact matches ($T_{c} = 1.0$), (2) the number of *bioactively similar* matches ($0.85< T_{c} < 1.00$) and (3) the overall success rate presented as the average Tanimoto similarity between predicted and true sequences (a series of fragments) over all the test molecules.

**Table 2:** Success rate over molecules on three test datasets

Datasets			
	Single	Double	All
Size			
	88,151	229,141	317,292
	9794	25,460	35,254
	9794	50,911	55,958
	74	73	74
Success rate			
Bi-LSTM			
	29.0%	27.9%	25.3%
	28.7%	10.5%	12.9%
	0.84	0.66	0.68
LSTM			
	22.9%	21.6%	19.4%
	29.7%	10.2%	12.5%
	0.82	0.62	0.64

^a^ Bioactively similar molecules

^b^ Average similarity

For the single reactant reactions, our bidirectional-LSTM model achieved an accuracy of 57.7% based upon the combined use of the first two criteria. The percentages of exact and bioactively similar matches were 29.0% and 28.7%, respectively. The average $T_{c}$ value between predicted and true sequences was 0.84. These results demonstrate that our machine predicts single reactant reactions with high accuracy. For the double reactant reactions, the success rate of the exact matches (27.9%) was almost identical to that of the single reactant reactions. However, the success rate of highly similar predictions deteriorated to 10.5% from 28.5%. For the combined set, 25.3% of predictions were accurate, and 12.9% of them were highly similar. Similarly, the average $T_{c}$ values dropped from 0.84 to 0.66 and 0.68 for datasets containing double and combined reactants.

One reason for the worse accuracy of the double and combined sets is that the “–” sign should be appropriately predicted. Another reason is the frequent occurrence of small molecules represented with a small number of MACCS keys in these datasets. In fact, 477 molecules represented with less than 7 MACCS keys appeared in 61822 different reactions. To be more specific, 3944 reactions contain a reactant represented with one of the seven MACCS keys described in Additional file 3: Figure S2. The number of unique structures corresponding to those keys was, however, only 29. Because such small and simple structures were dense in these datasets, wrongly predicted fragments contributed significantly (a value of zero in 1-bit cases) to the success rate.

Our result also demonstrates that the bidirectional LSTM-based model outperforms the unidirectional LSTM-based model. The success rates of exact matches become lower by about 6% for all the datasets consistently. This is possibly due to the fact that our MACCS key-based representation of a molecule does not depend on the order of keys. In other words, most information about molecules and chemical reactions are embedded into the co-occurences of keys.

### Global vs. local attention

We investigated the model performance on longer sequences with both global and local attention mechanisms. As a matter of fact, it may not be practical to use Luong’s global attention [56] for longer sequences since it has to attend to all words on the encoder side for each target word. For our dataset, the average length of a reactant–product pair is 74. To investigate if the local attention may improve prediction quality, we augment the dataset with more complex molecules, and perform experiments by applying both the local and global attention mechanisms. As shown in Table 3, the local attention mechanism yields *marginally better* results than the global attention mechanism for longer sequences, containing more than 100 fragments. However, the performance of the model trained with sequences up to 100 fragments do not improve with the local attention mechanism.

**Table 3:** Comparison of model accuracy based on selected attention mechanism on combined datasets

	Maximum reactant–product pair length			
	100	120	140	160
Dataset size	350 K	489 K	588 K	637 K
Testset size	55 K	79 K	96 K	105 K
Global ($T_{c} \ge 0.85$)	38.2%	37.5%	37.2%	37.2%
Local ($T_{c} \ge 0.85$)	38.3%	39.6%	38.6%	38.6%

### Comparison with existing models

We compared the prediction accuracy of our approach with other retrosynthetic prediction methods without considering reaction class labels because no prior reaction class information was provided to our model. Several recent reports summarized the prediction accuracy of various models [37, 62]. According to reproduced results presented by Lin et al. [37], Top-1 accuracy ranges from 28.3% (Liu et al. [34] LSTM model over the USPTO 50 K dataset) to 54.1% (Transformer model over the USPTO MIT dataset by Lin et al. [37]). In the most recent report by Tetko et al. [46], an augmented Transformer model has reached Top-1 accuracy of 53.5% trained with 100 times augmented USPTO-50 K dataset with beam size 10. Tetko et al. also trained their model using a fivefold augmented filtered USPTO-full training set, approx. 3.8M training data, and Top-1 accuracy is reported as 46.2%. These results are superior to our model’s predictive accuracy of perfect predictions, 29%, but inferior overall, 57.7%, if highly similar predictions are considered. As an alternative approach, Coley’s similarity-based model [63] achieved a Top-1 accuracy of 37.3% on the USPTO 50 K dataset.

### Fingerprint dependency

We trained our Bi-LSTM model with Extended Connectivity Fingerprints (ECFP, Morgan fingerprint as RDKit implementation) on the single reactant reaction dataset following the same preprocessing steps. We selected four types of ECFP with a fixed-length folding of 1024 and 2048 bits (nBits), and for a radius of 1 and 2. Compared to the MACCS key-based model, the models trained with the ECFP with a radius of 1 are showing higher percentage of exact matches (see Table 4). The highest percentage of exact matches is observed with the model with ECFP of radius 1 and nBits 2048. The percentage increased by 8.6% compared to the MACCS key-based model. However, the percentage of bioactively similar reactions, $T_{c}$ $\ge$ 0.85, 52%, remains comparable to that of the MACCS key-based model, 57.7%. These results suggest that ECFP with a radius of 1 provides better resolution than the MACCS keys.

**Table 4:** Comparison of model accuracy on single reactant reaction dataset using ECFP and MACCS keys

	MACCS	ECFP (Radius, nBits)			
		1,1024	1,2048	2,1024	2,2048
Encoder	126	1024	2016	1024	2048
Decoder	126	1024	2027	1024	2048
Ave. length	74	56	56	74	74
	Accuracy				
$T_{c} = 1.0$	29.0%	35.6%	37.6%	9.1%	10.1%
$T_{c} \ge 0.85$	57.7%	50.7%	52.0%	28.4%	30.0%
$\overline{T_{c}}$	0.84	0.80	0.80	0.66	0.66

However, the models trained with ECFP with a radius of 2 show dramatic decreases in accuracy of the exact matches, 9.1% and 10.1%. To identify the origin of such performance drop, we performed further analysis of fragments embedded in one bit of ECFP of various radii over the single reactant reaction dataset. The numbers of substructures associated with bits activated by atom environments of radius 1 and 2 are investigated (see Additional file 4: Figure S3). The set of regular expressions embedded in one token becomes more complex, suggesting that the recognition of chemical changes becomes more challenging in the same dataset. From the analysis, it is identified that the model becomes confused due to a high number of fragments embedded in one bit. With a radius of 1, each bit of ECFP contains 11 fragments on average. However, with a radius of 2, each bit includes 113 fragments on average, i.e., a large degeneracy of each bit. This large degeneracy makes the patterns of bits of chemical reactions highly complicated, which becomes too hard to learn. These analyses suggest that curating the optimal set of fragments and their proper representations is critical in improving retrosynthesis prediction quality.

### Learning behavior

**Fig. 4:**
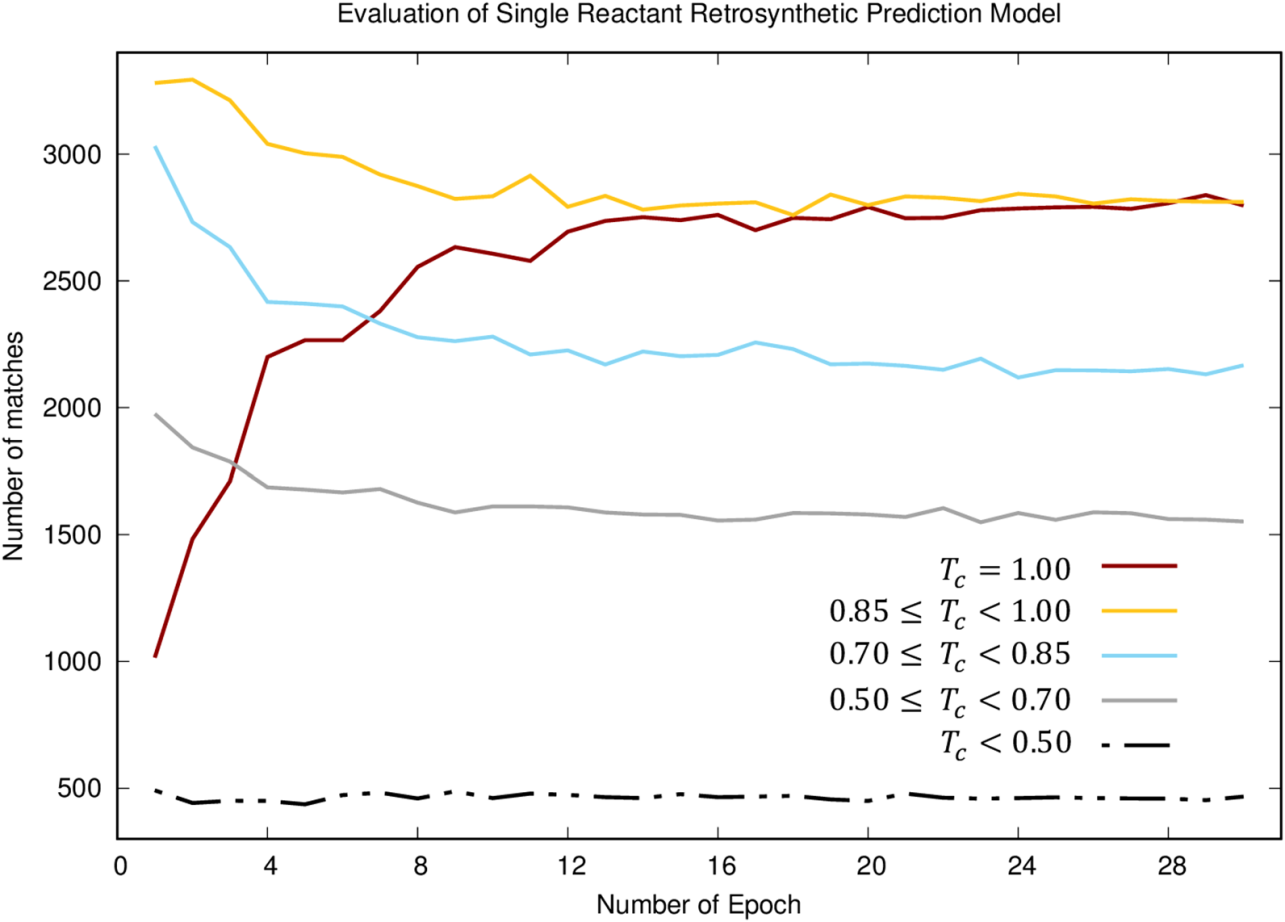
Number of matches at different ranges of similarity. T_c_ refers to Tanimoto similarity coefficient

To identify how our model learns the grammar of chemical reactions, the evolution of prediction accuracy with respect to threshold values along training epoch for the single reactant validation set is illustrated in Fig. 4. In particular, it is demonstrated that the network successfully learned reaction rules by capturing the alterations of molecules at a substructural level. The number of exact matches ($T_{c} = 1.0$) increased rapidly during the first 10 epochs. After 20 epochs, the value became almost tripled. The likelihood of making a better prediction for each fragment becomes higher during training. This is a clear indication of successful training. The improvement in exact matches appears to be a result of the respective declines in non-exact matches except extremely bad predictions ($T_{c} < 0.50$). The quality of bad predictions (ca. 5% of the validation set) did not improve probably due to the insufficient information, complexity, and noise contained in the data. This observation similarly repeated for all the other datasets.

### Similarity measure dependency

As an extension of our Tanimoto-based analyses, the effects of using other similarity metrics on our model’s accuracy is investigated. We select the Sörensen–Dice similarity as a special case of the Tversky index, and three asymmetric Tversky variants that include $\alpha$ and $\beta$ parameters. As illustrated in Additional file 5: Figure S4, we find that the model performance remains independent regardless of the choice of similarity metric. The number of similar molecules, however, changes across different regions based on how similarity is quantified. The Sörensen–Dice similarity behaves in a similar way as Tversky index when parameters $\alpha$ and $\beta$ are 0.1 and 0.9, respectively. Predicted sequences make larger contributions to their similarity to true sequences with smaller values of $\alpha$.

### Examples of retrosynthetic predictions

**Fig. 5:**
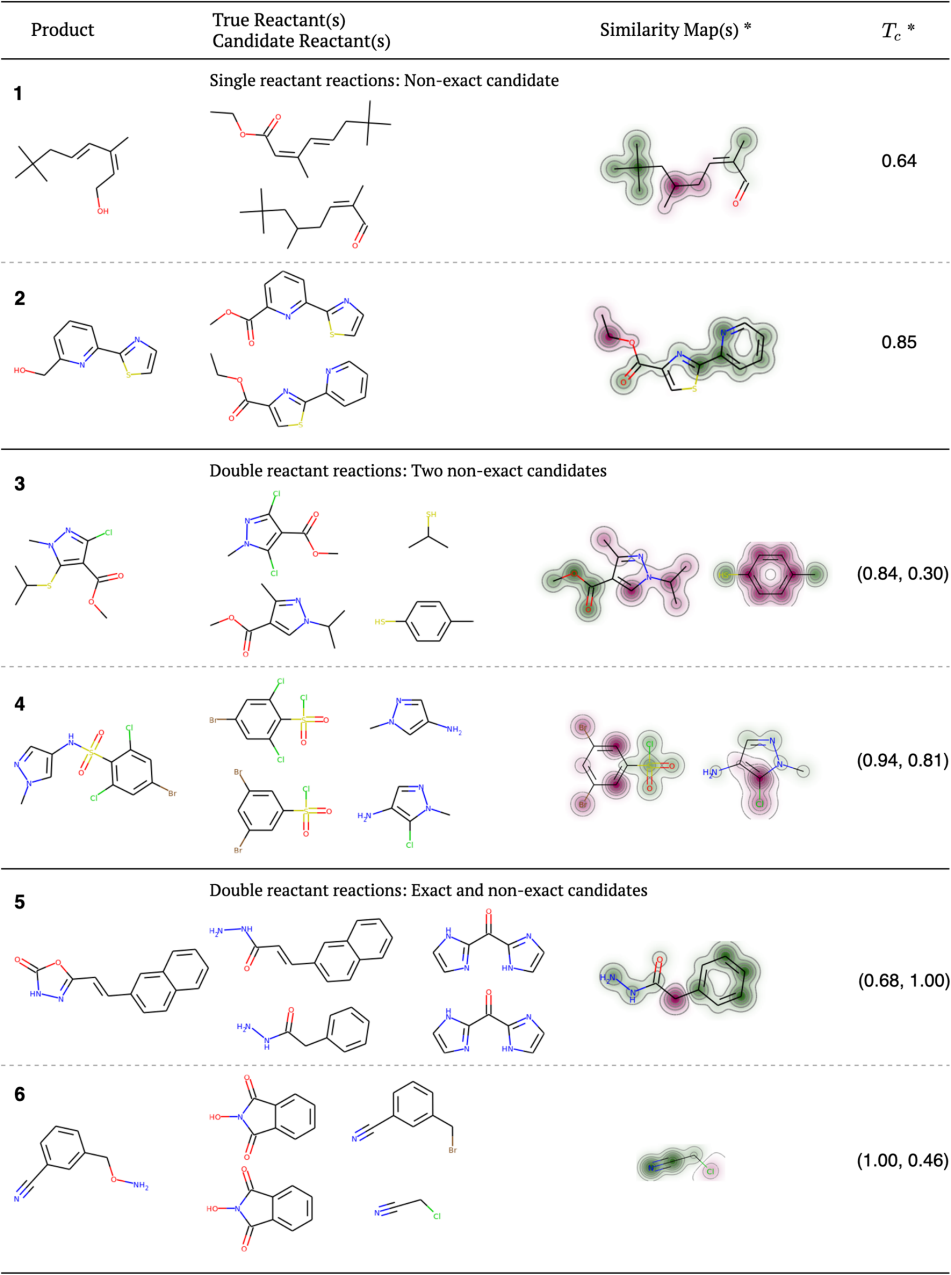
Non-exact candidates varied in their degree of similarity. *Similarity score calculations and similarity maps using the Morgan fingerprints and the Tanimoto metric are shown. Colors indicate atom-level contributions to the overall similarity (green: increases similarity score, red: decreases similarity score, uncolored: has no effect

In this study, we assumed that candidate reactants with $T_c>0.85$ are similar enough to their true counterparts. To validate this assumption, we assessed the quality of candidate reactants by comparing them with true reactants. We investigated whether the following factors were correct: functional group interconversion (FGI) or bond disconnection, reactive functional group, and core structure. The accuracy of side-substituents is regarded as less significant for matching the reactants’ functionality, especially when they are simple alkyls. Randomly chosen predictions exemplifying possible prediction cases are presented in Fig. 5. Similarity maps are presented to visualize similarities between candidates and true reactants.

Reaction 1 resulted in a reactant where the main chain composed of eight carbons, and an $\alpha$,$\beta$-unsaturated aldehyde group in the correct position was derived accurately (Fig. 5). Although an ester was expected rather than an aldehyde, an aldehyde reduction could also provide the same target alcohol. This indicates that our prediction identified the functional group interconversion correctly. On the other hand, one olefin was missing and the position and number of two methyl groups out of four were misinterpreted. In reaction 2, aside from the location of an ester group, core heterocyclic rings, pyridine and thiazole, and their connections were accurately generated. In the true reactant, a methyl ester group was attached to C6 of pyridine, whereas the ethyl ester group was attached to C4 of the thiazole ring in our candidate. If the position of the ethyl ester group was accurate, it would require a single-step reduction to obtain alcohol group. In reaction 3, the core structure of pyrazole ring and its methyl ester group were predicted accurately. However, there was no chloride, one of the reactive functional groups, and substituents on the pyrazole ring as well as structure of thiol were misinterpreted.

The result of reaction 4 showed that our model correctly predicted the core structures, bond disconnections, and reactive functional groups. However, the number and position of halides were wrong. In the case of reaction 5, one reactant was predicted precisely, but the other was partially incorrect. In wrongly predicted candidate, a (phenyl)methyl group appeared instead of a (2-naphthyl)vinyl group, but the reactive functional group, acylhydrazine, was correctly produced. The result of reaction 6 revealed the exact match for* N*-Hydroxyphtalimide as a precursor for* O*-hydroxylamine. However, the structure of the alkyl halide lacked a phenylene group. The core structure estimation failed to a great extent for this reaction. On the other hand, the reactive functional groups and bond disconnection are suggested correctly.

The quantitative summary of the assessment above is given in Table 5. The three criteria: functional group interconversion or bond disconnection, core structure, and reactive functional group are weighted equally. They are utilized to form a chemically reasonable score along with similarity scores. The evaluation was carried out by following procedure. First, we identified less significant parts of candidate molecules by comparing them with the product and true reactants. Second, core structures were identified; true reactants were separated into fragments, e.g., functional group, chain, ring. Afterwards, each fragment of a candidate molecule was evaluated against fragments found in second step in terms of the core structure, type and positions of side-substituents in an equally weighted manner. Finally, equal weight was given to the correctness of fragments’ positions within candidate reactants. Concerning the core structure, the longest chain of carbons and/or a ring, either of which may possess heteroatoms such as O, N, S, were taken into account together with important side-substituents and their positions. Because functional group interconversion or bond disconnection as well as reactive functional groups are the most significant factors of retrosynthetic analysis, the correct positions of reacting sites are scored strictly as true/false values corresponding to 1 and 0, respectively. We scored each candidate reactant individually and averaged the results to obtain a final score for each criterion.

It is noticeable that our model correctly predicted functional group interconversion or bond disconnection of all six reactions. Except for reaction 3, reactive functional groups are correctly reflected. We observe that prediction errors that affect the score are mainly associated with core structures. We applied this knowledge-based scoring strategy to a more specific set containing ten randomly chosen reactions where candidate reactants, on average, lies within bioactively similar region (T_c_ = 0.87) (Additional file 6: Figure S5A, Additional file 7: Figure S5B and Additional file 8: Table S2). The results clearly show that our model is highly accurate in predicting functional group interconversion or bond disconnection as well as reactive functional group for bioactively similar reactant candidates. A similar argument can also be made regarding the prediction errors, since they mainly originate from core structures.

**Table 5:** Summary of quality assessment of candidate reactants

Reaction number	FGI or bond disconnection^a^	Core structure^b^	Reactive functional group	Avg.^c^	T_c_^d^
1	1.00	0.33 (2/3 fragments)	1.00	0.78	0.64
2	1.00	0.67 (1/3 fragment’s positions)	1.00	0.89	0.85
3	1.00	0.69 (C1 = 0.88, fragment’s side subst.; C2 = 0.5, 1/2 fragments)	0.50 (1 for thiol, 0 for chloride)	0.73	0.57
4	1.00	0.96 (C1 = 0.92, position of side subst.; C2 = 1.0, Cl is omitted)	1.00	0.99	0.87
5	1.00	0.83 (C1 = 0.67,1/3 fragments; C2 is exact)	1.00	0.94	0.84
6	1.00	0.67 (C1 is exact; C2 = 0.33, 2/3 fragments)	1.00	0.89	0.73

^a^ The functional group interconversion (FGI) or bond disconnection and reactive functional group columns represent the correctness in a True(1)/False(0) fashion

^b^ The core structure column presents the averaged accuracy of the core structures of candidate molecules by capturing the correctness of core structures themselves as well as the type and positions of side-substituents. The source of errors are given inside the parenthesis e.g., *“C2=0.33, 2/3 fragments”* implies that the accuracy of candidate reactant 2 is 0.33 because 2 out of 3 fragments are wrongly predicted. C1: Candidate 1, C2: Candidate 2

^c^ The average of the three criteria

^d^ The averaged T_c_ values of candidate reactants

The chemical inspection of reactions indicates that average similarity scores and knowledge-based scores are closely related. Our scoring approach offers a clear idea about the quality of candidate reactants and similarity scores are in good agreement with those manually inspected. Similarity measurements yield lower scores than knowledge-based scores possibly due to the inclusion of side chains and geometrical factors (more detailed topological exploration is provided by Morgan fingerprint). Although the interpretation of the similarity score is rather difficult to assess objectively, it can be used for assessing the quality of retrosynthetic predictions. Higher similarity scores indicate that the desired molecules are more synthetically accessible according to the rules of organic chemistry.

### Characteristics of our model

The key advantage of our word-based MACCS keys model over the character-based SMILES methods is that the network needs to learn relatively simpler grammatical rules: ascending order and co-occurence of keys, to yield meaningful results. In the SMILES-based methods, a network has to comprehend not only the complicated grammar of SMILES but also the canonical representation to predict synthetically correct sequences. As summarized by Liu et al. [34], the difficulty of learning the syntactic structure of SMILES notation possibly causes problematic outcomes such as invalid SMILES strings. In general, existing character-based models suffer from the generation of literally invalid, literally valid but chemically unreasonable, or literally and chemically valid but unfeasible candidates. We avoided this problem by projecting the SMILES representation of a molecular structure into a substructural domain. Our approach can be an effective solution to these technical problems at a fundamental level.

In general, the likelihood of making correct retrosynthetic predictions remains rather low. Indeed, the accuracy of retrosynthetic planning tasks is twice as much lower than the level of accuracy achieved at forward reaction prediction tasks [17, 27, 31]. This is especially true assuming that several possible synthetic routes are available for the forward reaction. It is worth noting that the content of the dataset used in the reverse mapping, could also be responsible for the network’s behavior [62]. Mapping a reactant from a reactant domain to a product domain and then reversing it does not necessarily produce the original reactant considering the level of abstraction used to describe the molecules in our dataset. There is a chance that the presence of one-to-many mappings from a product to a reactant domain may create confusion during the learning process. Equipped with these observations, a simple idea is adopted to assure a stronger pairwise functional relationship between the domains. To achieve this, we identified all one-to-many mappings and collapsed them into an injective mapping (see Fig. 3, "Reaction dataset curation" section) by selecting the molecule with the shortest sequence length (presumably the reactants with the lowest level of structural complexity).

Notably, our model yields robust predictions. For each independent run of the same input molecule, our model gives the same output consistently. This robustness of our model may be due to the low complexity and good interpretability of our molecular descriptor. Generally, retrosynthetic models have employed the top-N accuracy score to assess overall model performances [11, 34–37, 45, 63]. However, as recently discussed by Schwaller [38], top-N accuracy score may not be an adequate metric for assessing retrosynthetic models because with each suggestion, the model tends to yield expected answers from the dataset rather than making chemically more meaningful predictions. Although MACCS keys have been criticized for their poor performance on similarity benchmarks [64], an advantage of such descriptor is that there is an one-to-one correspondence between a bit and a substructure compared to fingerprints obtained by an exhaustive generation algorithm followed by a hashing procedure. Thus, MACCS keys were a natural choice to test the proof-of-concept level of our translation methodology.

The diversity of reactant candidates is one of the important aspects of a retrosynthesis prediction. In the recently published paper [46], the diversity of the reactant candidates is discussed within the context of top-5 performance analysis. One of the goals of a retrosynthetic model is to obtain multiple precursor suggestions, and the top-N approach may suggest other probable reactant candidates. Our model is robust in terms of the number of predictions made, i.e., always yields the identical prediction, resulting in an absence from the top-N concept. However, our model has a certain level of flexibility. Since the model predicts fingerprints instead of the exact structures, multiple structures can be retrieved for a predicted sequence. We verify that the average number of molecules represented with the same modified (126-keys) MACCS keys are three within 154 million compounds in PubChem. In other words, we could find three valid reactant candidates on average using PubChem. This leads to a flexible interpretation because choices between reaction candidates enable us to use chemical expertise and intuition.

**Table 6:** Success rate of retrieving a reactant candidate from the PubChem database

Tanimoto coefficient	Ratio (%)	No. of discrepant keys
1.00	62	None
$\ge$.97	70	1
$\ge$.94	91	2
$\ge$.90	100	3 or 4

Average length of a molecule in PubChem DB is 42. Test set size = 21,827, PubChemDB size is approximately 154 M

By design, our model predicts the MACCS keys representations rather than SMILES strings. Converting predicted sequences of structural fingerprints to valid molecules requires a dictionary to look up the reactant candidates that match the fingerprint. Fortunately, for MACCS keys, the reference SMARTS value of any bit is preserved during translation. Unlike hash-based fingerprints, there is always a one-to-one correspondence between a key and its definition. We, therefore, take advantage of using a fingerprint built upon the predefined substructures and constructed a lookup table using the USPTO [49] and PubChem [65] databases to retrieve the molecules that match with predicted MACCS keys. If a perfect match is not found from the table, the closest match is selected as the candidate of a real molecule. Each object within the lookup table contains SMILES, MACCS keys, and “language representation” in our retrieval mechanism. A query is sent based on the “language representation”.

We investigated the success rate of retrieving a reactant candidate within the PubChem database (Table 6). More than 20 K medium length reactant predictions were compared with 154 million molecules of the database. Sixty-two percent of predictions matched with the existing molecules. The success rate increased to 91% when up to 2 key difference was allowed. Considering the average length of keys, 42, this difference corresponds to T_c_ of 0.94, which is reasonably high enough. Also, the maximum number of discrepant keys is four, corresponding to a T_c_ of 0.9. In other words, all predicted reactants were successfully retrieved from the database with up to 4 discrepant keys, T_c_ > 0.9. In summary, these results demonstrate that our approach is practical enough because all predicted reactants could find the exact molecules or highly similar molecules with a T_c_ threshold of 0.94.

**Fig. 6:**
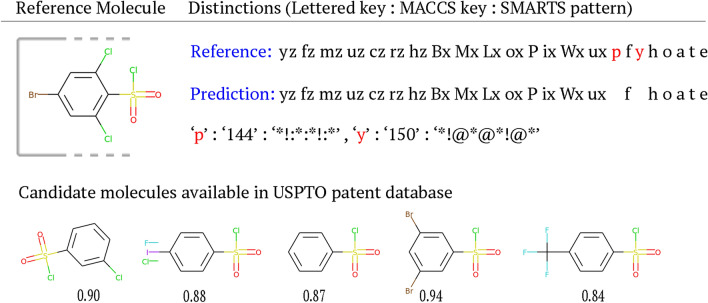
A detail look at the example 4 in Fig. 5. Similarity scores are shown using circular fingerprint (Morgan) with radius 2 and the Tanimoto metric. Distinct fragments are shown as SMARTS patterns [67]

Figure 6 depicts the seven candidates for the first reactant of the fourth reaction in Fig. 5 retrieved from the USPTO reaction dataset. All of the seven candidates are associated with different reactions in the database. The MACCS key representation of the retrieved molecules are identical. This implies that it is possible to find more than one match corresponding to the predicted sequence. These closely related analogs can be ordered by computing the Tanimoto coefficients using path-based or circular fingerprints as they will be different for the same set. For this purpose, we used the circular fingerprint [66] with radius 2 as a bit vector. We selected the molecule with the highest similarity value among candidates as our final result.

## Conclusion

We developed a sequence-to-sequence NMT model to extract the reaction rules of a chemical reaction automatically by learning the relationships at substructural level. By constructing an abstract language with a small size fixed-length vocabulary of non-zero elements of MACCS keys, three conceptual problems are addressed and resolved jointly: (1) erratic predictions: SMILES-based representation makes model outcomes prone to error, (2) synthetic availability: predicted molecules may not be synthetically accessible, and (3) top-N accuracy metric: suggestions made by the model may vary by model run. The comparison and quality inspections showed that our method successfully produced candidate reactants within a region 0.90 < T_c_ ≤ 1.00, achieving a high level of overall accuracy, particularly at functional group interconversion or bond disconnections and reactive functional groups. We believe that this proposed approach has a high potential for broad applications in organic chemistry. For the future version, it is essential to develop a better defined structural key suitable for reaction prediction purposes.

## Supplementary Information

**Additional file 1.** Dictionary Data. MACCS keys assignments. The set contains the assignments of letters to MACCS keys and list of used keys is presented.

**Additional file 2: Figure S1.** Sentence length distribution. Distribution profile of product-reactant pairs.

**Additional file 3: Figure S2. ** 1-bit keys. Examples to molecules that are represented with only one bit.

**Additional file 4: Figure S3.** Fingerprint dependency. Comparison of model accuracy using EFCP and MACCS.

**Additional file 5: Figure S4.** Similarity Measure Dependency. Effect of similarity metric type on model performance.

**Additional file 6: Figure S5A.** Bioactively similar reactions. Depictions of ten bioactively similar reactant candidates (1–5).

**Additional file 7: Figure S5B.** Bioactively similar reactions. Depictions of ten bioactively similar reactant candidates (6–10).

**Additional file 8: Table S1.** Hyperparameter settings. Hyperparameter settings for the best model.** Table S2.** Scoring of bioactively similar reactions. Assessment of candidate reactants lie in bioactively similar region.

## Data Availability

The datasets supporting the conclusions of this article are available via https://github.com/knu-chem-lcbc/fragment_based_retrosynthesis repository.
