# Supplementary material for: Substructure-based neural machine translation for retrosynthetic prediction
Source: J Cheminform. 2021 Jan 11;13:4. doi: 10.1186/s13321-020-00482-z (PMC7802345; doi:10.1186/s13321-020-00482-z)
Supplement: Supplementary file 1 — Additional file 1. Dictionary Data. MACCS keys assignments. The set contains the assignments of letters to MACCS keys and list of used keys is presented. [file 13321_2020_482_MOESM1_ESM.pdf]

RESEARCH

Substructure-based Neural Machine Translation for Retrosynthetic Prediction

Umit V. Ucak[1], Taek Kang[2], Junsu Ko[3*] and Juyong Lee[1*]

*Correspondence:
junsuko@arontier.co;
juyong.lee@kangwon.ac.kr
[3]Arontier co., Seoul, South Korea
[1]Division of Chemistry and Biochemistry, Department of Chemistry, Kangwon National University, Chuncheon, South Korea
Full list of author information is available at the end of the article

Abstract

Keywords: retrosynthesis planning; machine neural translation; seq-to-seq; attention

Additional Files as Text.

Please find the supporting materials as **text data** [**key : value**] **pairs** within the "Additional Files" section of the BMC article.

Author details
[1]Division of Chemistry and Biochemistry, Department of Chemistry, Kangwon National University, Chuncheon, South Korea. [2]Center for Neuro-Medicine, Korea Institute of Science and Technology, Seoul, South Korea. [3]Arontier co., Seoul, South Korea.

References
Additional Files

Additional file 1 — Dictionary Data.

Title of data : MACCS keys assignments
File format : Plain Text.
Description of data : The set contains the assignments of letters to MACCS keys and list of used keys is presented below.
{1: 0, 2: 0, 3: 0, 4: 0, 5: 0, 6: 0, 7: 0, 8: 'Fz', 9: 0, 10: 0, 11: 'Mz', 12: 0, 13: 'Pz', 14: 0, 15: 0, 16: 'Bz', 17: 'Wz', 18: 0, 19: 'Cz', 20: 0, 21: 0, 22: 'Rz', 23: 0, 24: 'pz', 25: 'Lz', 26: 'Vz', 27: 0, 28: 0, 29: 0, 30: 0, 31: 0, 32: 'Iz', 33: 'Oz', 34: 'Uz', 35: 0, 36: 'gz', 37: 'Sz', 38: 'tz', 39: 0, 40: 0, 41: 'Tz', 42: 0, 43: 'Dz', 44: 0, 45: 'Gz', 46: 0, 47: 'vz', 48: 0, 49: 0, 50: 'Hz', 51: 'yz', 52: 'nz', 53: 'Ez', 54: 'bz', 55: 'fz', 56: 0, 57: 'oz', 58: 'mz', 59: 0, 60: 'uz', 61: 'cz', 62: 'yx', 63: 0, 64: 0, 65: 'N', 66: 'Cx', 67: 'rz', 68: 'Yz', 69: 'wz', 70: 0, 71: 'lz', 72: 'iz', 73: 'hz', 74: 'Rx', 75: 'wx', 76: 'Az', 77: 'Ax', 78: 'Nz', 79: 'vx', 80: 'px', 81: 'Bx', 82: 'Yx', 83: 'F', 84: 'az', 85: 'mx', 86: 'Tx', 87: 0, 88: 'Mx', 89: 'Px', 90: 'Ux', 91: 'Dx', 92: 'bx', 93: 'cx', 94: 'Ox', 95: 'gx', 96: 'M', 97: 'rx', 98: 'R', 99: 'dz', 100: 'V', 101: 'sx', 102: 'Lx', 103: 0, 104: 'Sx', 105: 'ex', 106: 'ox', 107: 0, 108: 'Fx', 109: 'fx', 110: 'G', 111: 'W', 112: 'P', 113: 'dx', 114: 'Vx', 115: 'ez', 116: 'Gx', 117: 'U', 118: 'C', 119: 'sz', 120: 'A', 121: 'm', 122: 'S', 123: 'Y', 124: 'ix', 125: 0, 126: 'E', 127: 'D', 128: 'lx', 129: 'tx', 130: 'Wx', 131: 'Nx', 132: 'hx', 133: 'B', 134: 0, 135: 'nx', 136: 'ux', 137: 'd', 138: 'ax', 139: 'Ex', 140: 'Hx', 141: 'Ix', 142: 'g', 143: 'L', 144: 'p', 145: 'b', 146: 'O', 147: 'I', 148: 'f', 149: 'H', 150: 'y', 151: 'T', 152: 'v', 153: 'u', 154: 'c', 155: 'w', 156: 'n', 157: 'r', 158: 'l', 159: 'h', 160: 's', 161: 'i', 162: 'o', 163: 'a', 164: 't', 165: 'e', 166: 0}
