# Supplementary material for: Substructure-based neural machine translation for retrosynthetic prediction
Source: J Cheminform. 2021 Jan 11;13:4. doi: 10.1186/s13321-020-00482-z (PMC7802345; doi:10.1186/s13321-020-00482-z)
Supplement: Supplementary file 7 — Additional file 7: Figure S5B. Bioactively similar reactions. Depictions of ten bioactively similar reactant candidates (6–10). [file 13321_2020_482_MOESM7_ESM.pdf]

RESEARCH

Substructure-based Neural Machine Translation for Retrosynthetic Prediction

Umit V. Ucak[1], Taek Kang[2], Junsu Ko[3*] and Juyong Lee[1*]

*Correspondence:
junsuko@arontier.co;
juyong.lee@kangwon.ac.kr
[3]Arontier co., Seoul, South Korea
[1]Division of Chemistry and Biochemistry, Department of Chemistry, Kangwon National University, Chuncheon, South Korea
Full list of author information is available at the end of the article

Abstract

Keywords: retrosynthesis planning; machine neural translation; seq-to-seq; attention

Additional Files as Figures.

Please find the supporting materials as **figures** within the "Additional Files" section of the BMC article.

Author details
[1]Division of Chemistry and Biochemistry, Department of Chemistry, Kangwon National University, Chuncheon, South Korea. [2]Center for Neuro-Medicine, Korea Institute of Science and Technology, Seoul, South Korea. [3]Arontier co., Seoul, South Korea.

References

Additional Files

Additional File 7 : Figure S5B

File name : Supplementary Figure S5B
Title of data : Bioactively similar reactions
File format : Standard Latex figure, formatted as PNG.
Description of data : Depictions of ten bioactively similar reactant candidates (6-10)

Figure S5B: Ten reactions lie in the bioactively similar region used to assess the quality of retrosynthesis (6-10).
