# Supplementary material for: Substructure-based neural machine translation for retrosynthetic prediction
Source: J Cheminform. 2021 Jan 11;13:4. doi: 10.1186/s13321-020-00482-z (PMC7802345; doi:10.1186/s13321-020-00482-z)
Supplement: Supplementary file 8 — Additional file 8: Table S1. Hyperparameter settings. Hyperparameter settings for the best model. Table S2. Scoring of bioactively similar reactions. Assessment of candidate reactants lie in bioactively similar region. [file 13321_2020_482_MOESM8_ESM.pdf]

RESEARCH

Substructure-based Neural Machine Translation for Retrosynthetic Prediction

Umit V. Ucak[1], Taek Kang[2], Junsu Ko[3*] and Juyong Lee[1*]

[*]Correspondence:
junsuko@arontier.co;
juyong.lee@kangwon.ac.kr
[3]Arontier co., Seoul, South Korea
[1]Division of Chemistry and Biochemistry, Department of Chemistry, Kangwon National University, Chuncheon, South Korea
Full list of author information is available at the end of the article

Abstract

Keywords: retrosynthesis planning; machine neural translation; seq-to-seq; attention

Additional Files as Tables.

Please find the supporting materials as **tables** within the "Additional Files" section of the BMC article.

Author details
[1]Division of Chemistry and Biochemistry, Department of Chemistry, Kangwon National University, Chuncheon, South Korea. [2]Center for Neuro-Medicine, Korea Institute of Science and Technology, Seoul, South Korea. [3]Arontier co., Seoul, South Korea.

References

Additional Files

Additional File 8 : Table S1

File name : Supplementary Table S1
Title of data : Hyperparameter settings
File format : Latex table.
Description of data : Hyperparameter settings for the best model.

Table S1: Hyper-parameter space and hyper-parameters for the best model.

Parameter	Possible Values	Best Model Parameters
RNN Cell Type	LSTM or Bi-LSTM	Bi-LSTM (Encoder & Decoder)
Number of Layers	2, 4, or 6	2
Number of units	500,1000, 2000	2000
Learning Rate	0.1 - 8	4
Decay factor	0.50 - 0.90	0.85
Dropout	0.1 - 0.5	0.1
Type of Attention		Luong's global attention mechanism

Additional File 8 : Table S2

File name : Supplementary Table S2
Title of data : Scoring of bioactively similar reactions
File format : Latex table.
Description of data : Assessment of candidate reactants lie in bioactively similar region

Table S2: The quantitative summary of the assessment of the specific set containing ten reactions where the candidate reactants lie in the bioactively similar region.

Reaction Number	FGI or Bond Disconnection[1]	Core Structure[2]	Reactive Functional Group	Avg.[3]	T_c[4]
1	1.00	0.98 (C1=1.00; C2=0.95, 6/5 #C in Alkyl)	1.00	0.99	0.87
2	1.00	0.83 (C1=1.00; C2=0.67, 1/3 fragments)	1.00	0.94	0.81
3	1.00	1.00	1.00	1.00	0.84
4	1.00	0.75 (C1=1.00; C2=0.50, 1/2 fragments)	1.00	0.92	0.87
5	1.00	0.79 (C1=0.75, 1/2 fragment's position; C2=0.83, 1/3 fragment's position)	1.00	0.93	0.86
6	1.00	0.88 (C1=0.75, 1/2 fragment's position; C2=1.00)	1.00	0.96	0.94
7	1.00	0.96 (C1=0.97, 5/6 #C in ring ; C2=0.94, 5/4 #C in Alkyl)	1.00	0.99	0.91
8	1.00	1.00	1.00	1.00	0.87
9	1.00	1.00	1.00	1.00	0.83
10	1.00	0.97 (C1=1.00; C2=0.94, position of side subst.)	1.00	0.99	0.85

[1] The functional group interconversion (FGI) or bond disconnection and reactive functional group columns represent the correctness in a True(1)/False(0) fashion.
[2] The core structure column presents the averaged accuracy of the core structures of candidate molecules by capturing the correctness of core structures themselves as well as the type and positions of side-substituents. The source of errors are given inside the parenthesis e.g., "*C2=0.33, 2/3 fragments*" implies that the accuracy of candidate reactant 2 is 0.33 because 2 out of 3 fragments are wrongly predicted. C1: Candidate 1, C2 : Candidate 2.
[3] The average of the three criteria.
[4] The averaged T_c values of candidate reactants.
